# Hippocrates Asserted That Temporomandibular Joint Dislocation Could Be Fatal if Not Reduced

**DOI:** 10.7759/cureus.92963

**Published:** 2025-09-22

**Authors:** Kazuya Yoshida

**Affiliations:** 1 Department of Oral and Maxillofacial Surgery, National Hospital Organization, Kyoto Medical Center, Kyoto, JPN

**Keywords:** hippocrates, historical perspective, intracranial complications, mandibular condyle, maxillofacial surgery, middle cranial fossa, neurological sequelae, superior dislocation, temporomandibular joint dislocation

## Abstract

Hippocrates (ca. 460-370 BC), often referred to as the *Father of Medicine*, described several aspects of dentistry and maxillofacial surgery, including techniques for reducing temporomandibular joint (TMJ) dislocation. In his writings, he noted that untreated TMJ dislocation could be fatal within 10 days, attributing this outcome to systemic complications such as fever, coma, and gastrointestinal disturbances. Although this account has traditionally been considered controversial and was largely dismissed by subsequent physicians, it gains potential credibility when reconsidered in light of rare superior dislocations of the mandibular condyle into the middle cranial fossa. Contemporary reports have documented neurological sequelae and even fatalities associated with such superior TMJ dislocations. This perspective revisits Hippocrates’ original description, contrasts it with later medical interpretations, and suggests that his observation may represent one of the earliest clinical accounts of traumatic superior condylar dislocation with intracranial involvement.

## Editorial

Temporomandibular joint (TMJ) dislocation is a relatively common presentation in oral and maxillofacial surgery [1,2]. While anterior dislocations are most common, posterior, lateral, and superior dislocations have been described, with superior dislocation being exceedingly rare [1]. The Hippocratic method of reduction for anterior TMJ dislocation, a manual maneuver, remains widely practiced even today [2].

**Figure 1:**
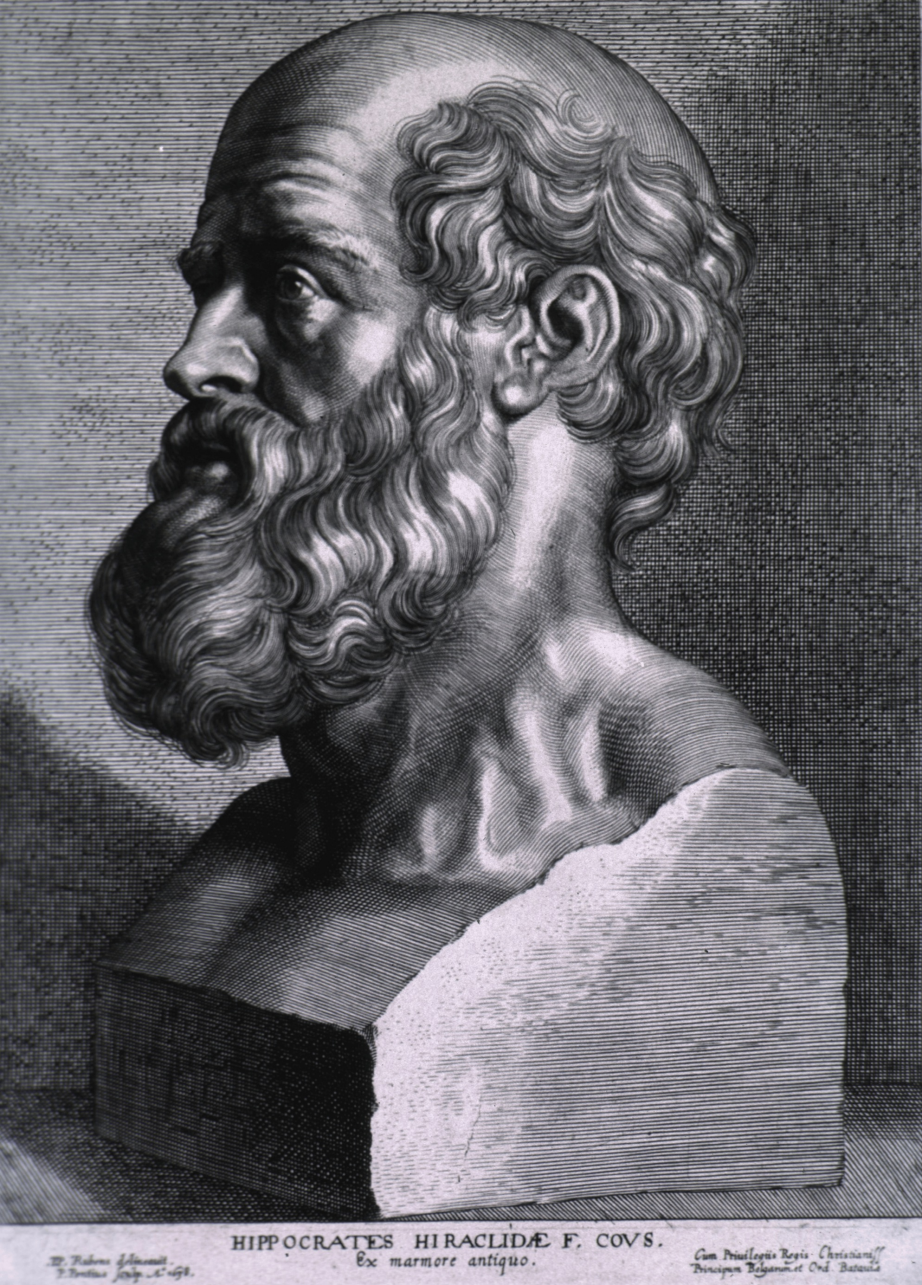
Hippocrates of Kos (460-380 BC), engraving by Peter Paul Rubens, 1638. Source: Wikimedia Commons (commons․wikimedia․org/wiki/File:Hippocrates_rubens․jpg), original work in the public domain.

Hippocrates (ca. 460-370 BC), a Greek physician and philosopher, is considered one of the most outstanding scholars in medicine (Figure 1). Recognized as the *Father of Medicine*, Hippocrates earned respect and honor for his revolutionary principles and practice of medicine.

**Figure 2:**
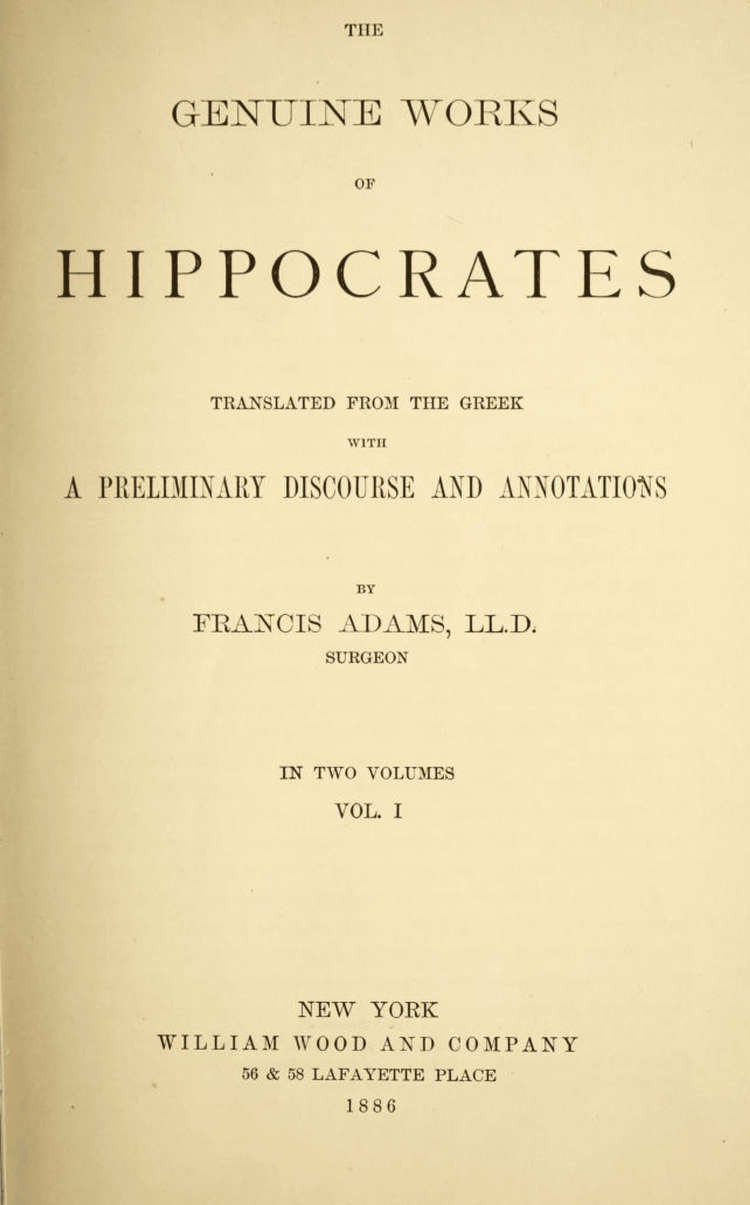
The genuine works of Hippocrates. Translated from Greek with a Preliminary Discourse and Annotations by Francis Adams [3]. Source: Internet Archive (https://archive.org/details/genuineworksofhi00tran/page/106/mode/2up), original work in the public domain.

Hippocrates’ collected works, *Hippocrates Corpus*, translated in the 19th century by Francis Adams [3], include detailed accounts of TMJ dislocation (Figure 2). The Hippocrates Corpus describes the reduction method and prognosis of TMJ dislocation [3] as follows:

“It is safer to operate with the patient laid on his back, and his head supported on a leather cushion well filled, so that it may yield as little as possible, but some person must hold the patient’ head.

If not reduced, the patient’s life will be in danger from continual fevers, coma attended with stupor (for these muscles, when disordered and stretched preternaturally, induce coma); and there is usually diarrhoea attended with bilious, unmixed, and scanty dejections; and the vomitings, if any, consist of pure bile, and the patients commonly die on the tenth day.”

The Hippocratic technique remains the most commonly recognized method for the manual reduction of anterior TMJ dislocations [1,2]. In this method, the physician places the thumbs laterally next to the molars while supporting the mandible with the other fingers, applying downward force followed by posterior pressure to reposition the joint [2]. Today, most practitioners associate the Hippocratic reduction method with a seated patient [1,2]. However, the original description specified the supine position, which was later revised in subsequent texts to allow either “seated or supine” positioning [4]. During Hippocrates’ era, warfare was prevalent, and injured soldiers or patients often required immediate treatment on the spot rather than being transported to a chair [4].

Of particular note, Hippocrates warned that if reduction was not performed, patients might develop fevers, stupor, diarrhea, vomiting of bile, and often die by the tenth day [3]. The assertion that unreduced TMJ dislocation could lead to death within 10 days was endorsed by Guy de Chauliac in 1363 and later by Ambroise Paré in 1575 [4]. Similarly, in 1719, Lorenz Heister upheld Hippocrates’ view, stating that failure to reduce a TMJ dislocation would result in the patient’s death [4].

**Figure 3:**
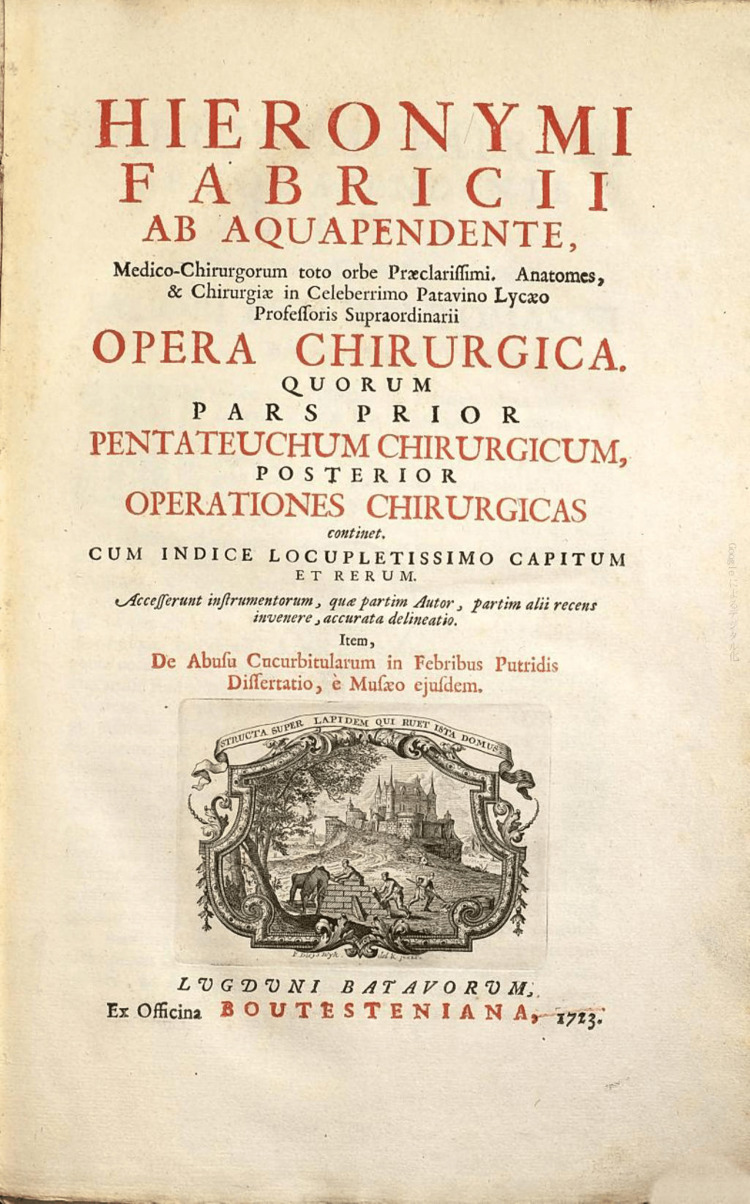
Opera Chirurgica by Fabricius ab Aquapendente H, 1723. Medico-chirurgicorum toto orbe praeclarissimi, anatomes & chirurgiae in celeberrimo Patavino Lycaeo professoris supraordinarii, Opera chirurgica: quorum pars prior pentateuchum chirurgicum, posterior operationes chirurgicas continent (The most illustrious surgeons throughout the world, professor extraordinary of anatomy and surgery at the renowned university of Padua, Surgical Works: the first part contains the pentateuch of surgery, the second part contains surgical operations) [5]. Source: Google Books (https://www.google.co.jp/books/edition/Hieronymi_Fabricii_ab_Aquapendente_Opera/jvxPZRRSwVYC?hl=ja&gbpv=1&dq=Opera+chirurgica+:+quorum+pars+prior+pentateuchum+chirurgicum,+posterior+operationes+chirurgicas+continent+by+Hieronymi+Fabricii+ab+Aquapendente.&pg=PA401&printsec=frontcover), original work in the public domain.

In 1723, Fabricius ab Aquapendente was the first to challenge Hippocrates’ prognosis, remarking that he had never observed fatal consequences from unreduced dislocations (Figure 3) [5]. Other physicians, such as Abraham Rees and Astley Cooper, likewise dismissed the claim because they had not encountered such cases [4]. In 1804, Boyer, followed by Abraham Rees in 1819, suggested that Hippocrates may have confused TMJ dislocation with trismus [4]. Consequently, Hippocrates’ assertion that an unreduced TMJ dislocation could lead to death within 10 days was omitted from later translations, presumably because subsequent physicians failed to confirm such outcomes [4].

Nevertheless, reconsidering Hippocrates’ account in the context of superior dislocations suggests that he may have accurately described a rare but devastating condition [1]. From a modern perspective, prolonged unreduced anterior dislocations are not life-threatening [2]. A long-standing or chronic TMJ dislocation is defined as one persisting for more than one month without reduction [2]. In a recent review of 229 cases drawn from 113 reports, the mean duration of dislocation was 11.9 months [2]. Closed reduction was successful in 49 patients (21.4%), while open reduction was required in 175 patients (76.4%) [2].

By contrast, superior dislocation of the mandibular condyle into the middle cranial fossa represents an entirely different clinical scenario [1]. A comprehensive review, combining electronic database and manual searches, identified 116 cases across 104 studies [1]. This condition is considered exceedingly rare due to protective anatomical mechanisms that normally prevent penetration of the cranial base by the condyle [1]. Specifically, even when a strong, upward-directed force applied to the chin is transmitted to the mandibular condyle, the structural features of the craniofacial region usually prevent penetration of the cranium [1]. The ratio between closed and open reduction procedures was the same within 7 days, but the frequency of closed reduction declined over time, and after 22 days, all cases required open surgery [1]. Notably, 80% of patients with complete intrusion of the condyle into the middle cranial fossa underwent open reduction [1].

The most common causes were motor vehicle accidents (50%), falls (20.7%), bicycle accidents (16.4%), assaults (3.4%), and collisions (1.7%) [1]. Neurological complications included loss of consciousness (19.8%), intracranial hematoma (17.2%), otorrhagia (14.7%), dural tear (14.7%), hearing loss (13.8%), and cerebrospinal fluid leakage (3.4%) [1]. Epidural hematomas were linked to laceration or rupture of the middle meningeal artery, whereas subdural hematomas were associated with injury to the posterior cerebral artery. Among the 116 cases reported, 25% were managed with closed reduction, 56.9% with open reduction, and 21.6% required craniotomy [1].

Forceful manual attempts to reposition an intruded condyle into the cranial fossa can result in stupor, vomiting, or even fatal brain injury, symptoms reminiscent of those described by Hippocrates. Indeed, four patients have died from related complications [1]. With modern diagnostic imaging, recognition of superior condylar dislocation is straightforward [1]. However, in Hippocrates’ era, without imaging, antibiotics, or neurosurgical treatment, the mortality rate would have been markedly higher.

It is therefore reasonable to speculate that Hippocrates’ account may have reflected acute superior dislocations associated with brain injury. His observations could represent the earliest documented recognition of the lethality of cranial penetration by the mandibular condyle. Later physicians, unfamiliar with this extremely rare pathology, may have dismissed his description as an error, leading to its removal in some translations.

In conclusion, Hippocrates’ statement that TMJ dislocation could be fatal if not reduced has long been disputed. While unreduced anterior dislocations are not fatal, his claim is consistent with cases of traumatic superior dislocation into the middle cranial fossa, which may indeed cause coma and death. This historical perspective underscores the importance of carefully interpreting classical medical texts in the context of rare but severe clinical entities. Hippocrates may have been the first physician to identify the potentially lethal consequences of superior TMJ dislocation.
